# Laboratory characterization of invasive *Haemophilus influenzae* isolates from Nunavut, Canada, 2000–2012

**DOI:** 10.3402/ijch.v75.29798

**Published:** 2016-01-12

**Authors:** Raymond S. W. Tsang, Y. Anita Li, Angie Mullen, Maureen Baikie, Kathleen Whyte, Michelle Shuel, Gregory Tyrrell, Jenny A. L. Rotondo, Shalini Desai, John Spika

**Affiliations:** 1 National Microbiology Laboratory, Public Health Agency of Canada, Winnipeg, Manitoba; 2 Centre for Immunization and Respiratory Infectious Diseases, Public Health Agency of Canada, Ottawa, Ontario; 3 Department of Health, Government of Nunavut, Iqaluit, Nunavut; 4 Provincial Laboratory for Public Health, Edmonton, Alberta, Canada

**Keywords:** *Haemophilus influenzae*, invasive strains, Nunavut, Canada

## Abstract

**Background:**

With invasive *Haemophilus influenzae* serotype b (Hib) disease controlled by vaccination with conjugate Hib vaccines, there is concern that invasive disease due to non-serotype b strains may emerge.

**Objective:**

This study characterized invasive *H. influenzae* (Hi) isolates from Nunavut, Canada, in the post-Hib vaccine era.

**Methods:**

Invasive *H. influenzae* isolates were identified by conventional methods at local hospitals; and further characterized at the provincial and federal public health laboratories, including detection of serotype antigens and genes, multi-locus sequence typing and antibiotic susceptibility.

**Results:**

Of the 89 invasive *H. influenzae* cases identified from 2000 to 2012, 71 case isolates were available for study. There were 43 serotype a (Hia), 12 Hib, 2 Hic, 1 Hid, 1 Hie, 2 Hif and 10 were non-typeable (NT). All 43 Hia were biotype II, sequence type (ST)-23. Three related STs were found among the Hib isolates: ST-95 (n=9), ST-635 (n=2) and ST-44 (n=1). Both Hif belonged to ST-124 and the 2 Hic were typed as ST-9. The remaining Hid (ST-1288) and Hie (ST-18) belonged to 2 separate clones. Of the 10 NT strains, 3 were typed as ST-23 and the remaining 7 isolates each belonged to a unique ST. Eight Hib and 1 NT-Hi were found to be resistant to ampicillin due to β-lactamase production. No resistance to other antibiotics was detected.

**Conclusion:**

During the period of 2000–2012, Hia was the predominant serotype causing invasive disease in Nunavut. This presents a public health concern due to an emerging clone of Hia as a cause of invasive *H. influenzae* disease and the lack of published guidelines for the prophylaxis of contacts. The clonal nature of Hia could be the result of spread within an isolated population, and/or unique characteristics of this strain to cause invasive disease. Further study of Hia in other populations may provide important information on this emerging pathogen. No antibiotic resistance was detected among Hia isolates; a small proportion of Hib and NT-Hi isolates demonstrated resistance to ampicillin due to β-lactamase production.

*Haemophilus influenzae* (Hi) is usually regarded as a strict human pathogen without any animal reservoir and can be divided into those encapsulated with a polysaccharide capsule and those non-encapsulated (1). Those with capsules can be divided into six capsular types called serotypes (a–f) using specific antisera raised against the unique chemical structure of each capsular type, and non-encapsulated strains are regarded as non-typeable (NT) (2). Of the six serotypes, type b (Hib) is the most important as it was a very common cause of serious invasive diseases. Before introduction of the Hib conjugate vaccine, most invasive *H. influenzae* diseases were caused by Hib (3,4) and most invasive Hib disease occurred in those under the age of 5 (5) causing serious invasive diseases such as meningitis, septicaemia, epiglottitis, pneumonia and septic arthritis. Population-based studies have also revealed very high incidence rates (500–700 cases per 100,000 population) of invasive *H. influenzae* disease among Indigenous children living in the Arctic (6,7). Since the introduction of vaccines against Hib in industrialised countries, a significant shift in the relative proportion of serotypeable strains other than Hib or NT strains involved in invasive diseases has been reported (8–10). In Nunavut, the Hib conjugate vaccine was introduced in 1997. Therefore, this study focused on the characteristics of invasive *H. influenzae* isolated from patients in Nunavut, Canada, in the post-Hib vaccine era. Nunavut is the largest and the most northern territory in Canada. It has a small population (estimated to be 36,585 as of July 2014) over a vast area of 1,877,787 km^2^. The objective of this study is to define the current strains involved in invasive *H. influenzae* diseases in northern Canada, which may lead to a discussion on potentially appropriate public health responses to an altered epidemiology involving mainly non-Hib strains in a population that may be more vulnerable to invasive *H. influenzae* disease.

## Materials and methods

### Case identification and isolates

Cases of invasive *H. influenzae* disease were identified according to the method employed in the International Circumpolar Surveillance Program on invasive bacterial diseases (11) and the case definition was further updated in 2009 (12).

The Provincial Laboratory for Public Health in Edmonton, Alberta, served as the main reference laboratory for Nunavut. Isolates were originally cultured at either the local hospitals or tertiary care facilities outside Nunavut, with most referral patients being transferred to the province of Alberta. At the Provincial Laboratory in Edmonton, cultures were identified and confirmed as *H. influenzae* and serotyped. Therefore, all isolates included in this study have been provided by the Provincial Laboratory for Public Health in Edmonton, Alberta.

### Cultures identification

All isolates were identified by standard biochemical tests (13), and biotyping was determined according to biochemical reactions to detect urease, indole and ornithine decarboxylase production in strains (14). Since NT-Hi are highly similar to *H. haemolyticus* (15), their identities were confirmed by 16S rRNA sequencing (16).

### Serotyping and detection of partial deletion of capsule synthesis genes (IS1016 and bexA)

This was done by the slide agglutination method using Difco antisera obtained from Becton Dickinson (Oakville, Ontario, Canada) and confirmed by PCR amplification of the capsule polysaccharide synthesis genes (17). Detection of IS*1016* and *bexA* deletion was accomplished as previously described (10).

### Multi-locus sequencing typing

Amplification of 7 housekeeping enzyme genes was done according to a published method (18), and assignment of sequence type (ST) was done through the *H. influenzae* MLST website (www.pubmlst.org/hinfluenzae/).

### Pulsed-field gel electrophoresis

Cultures were suspended in Tris-EDTA (TE) buffer (100 mM) to a turbidity of 0.5 using a Dade Behring MicroScan turbidity meter (Dade Behring, West Sacramento, California). Pulsed-field gel electrophoresis (PFGE) plugs were prepared from the cell suspension by mixing with 1.6% SeaKem Gold agarose (Mandel, Guelph, Ontario, Canada). Bacterial cells within the plugs were lysed with a 100 mM TE buffer containing 0.5 mg/ml proteinase K and 1% Sarkosyl at 50°C for 90 minutes. After a series of washes with water and TE buffer, the released bacterial DNA was digested with *Sma*I restriction enzyme (Invitrogen, Burlington, Ontario, Canada). PFGE was performed in a CHEF-DR III (Bio-Rad Laboratories, Mississauga, Ontario, Canada) using conditions described by St. Sauver et al. (19).

### Antibiotic susceptibility testing

Antibiotic susceptibility disk diffusion testing was conducted according to CLSI guidelines (20) using the following antibiotic disks from Oxoid (Nepean, Ontario, Canada): ampicillin 10 µg; chloramphenicol 30 µg; ceftriaxone 30 µg; trimethoprim–sulfamethoxazole 25 µg; amoxicillin–clavulanic acid 30 µg; cefaclor 30 µg; ciprofloxacin 5 µg; moxifloxacin 5 µg; clarithromycin 15 µg; azithromycin 15 µg; tetracycline 30 µg; levofloxacin 5 µg and meropenem 10 µg. Production of β-lactamase was determined using DrySlide Nitrocefin (Becton Dickinson).

## Results

### Number of invasive *H. influenzae* cases and incidence rates

During the 13-year period from 2000 to 2012, a total of 89 cases were identified and reported to public health, which ranged from a low of 3 cases in each of the years 2003 and 2008 to a high of 12 cases in 2001. The overall mean annual incidence rate was 22.3 cases per 100,000 population (range: 9.4–42.7 per 100,000).

Among the 89 cases, 55 were reported to be due to serotype a (Hia), 13 due to Hib, 2 due to serotype d (Hid), 1 due to serotype e (Hie), 2 due to serotype f (Hif), 11 due to NT-Hi and for 2 cases, the serotype information was missing. Twenty-seven of the 55 (49%) Hia cases were <1 year old; 23 cases (42%) were between the ages of 1 and 4 years old; 1 case was between 5 and 9 years old; and the remaining 4 cases were aged between 20 and 40 years old. The mean annual incidence rate of Hia disease was 13.7 per 100,000 population (range: 3.1–21.3 per 100,000). For those aged <1 year, the mean annual Hia incidence rate was 274.8 per 100,000 and for those aged between 1 and 4 years old, the mean annual Hia incidence rate was 61.2 per 100,000.

The gender distribution of the 89 invasive *H. influenzae* cases showed 49 males, 39 females and in 1 case, the gender information was not recorded. For Hia, 33 cases (60%) were males and 22 cases (40%) were females. For both Hib (5 males, 7 females; and 1 case with no gender information) and NT-Hi cases (6 males and 5 females), gender difference was not noted. The overall median age of all 89 cases was <1 year old, and for both Hia and Hib cases, their median ages were also <1 year old; while for the NT-Hi cases, their median age was 1 year old.

Seventy-eight (87.6%) of the 89 cases were Inuit and in the remaining 11 cases, there was no information on their ethnicity. For Hia, 49 (89.1%) of the 55 cases were Inuit, and in 6 cases, no ethnic information was available. There were a total of 6 deaths (5 were due to Hia and 1 due to NT-Hi) among the 89 cases with an overall case fatality ratio of 6.7%. However, for Hia cases, the case fatality ratio was 9.1%.

Seventy-one case isolates were available for analysis and they ranged from a low of 3 case isolates in each of the years 2003, 2008, 2009, 2010 and 2012 to a high of 11 case isolates in 2001. In addition, 4 cases in 2000, 2001 and 2002 provided duplicate isolates and in each case, the duplicate isolate from the same case provided identical results. Of the 18 cases without available culture, 12 were reported to be Hia, 1 each of Hib, Hic, Hid, NT and 2 without serotype information. Isolates from these 18 cases were either discarded without storage or lost during storage, and therefore, they were not available for further characterization.

### Serotype and biotype of invasive *H. influenzae*

Of the 71 case isolates, 60.6% or 43 belonged to Hia, 16.9% or 12 belonged to Hib and there were 2 each of Hic and Hif as well as 1 each of Hid and Hie. In addition, there were 10 case isolates that could not be serotyped by bacterial agglutination or PCR and were deemed to be non-encapsulated or NT. The yearly distribution of the different serotypes is found in Table I.

**Table I:** Serotype distribution according to year of isolation

	Number of cases (isolates) by serotype							
	
Year	a	b	c	d	e	f	NT	Total
2000	4 (5)	0	0	0	0	0	1	5 (6)
2001	6 (8)	2	0	0	0	0	3	11 (13)
2002	4 (5)	1	0	0	0	0	0	5 (6)
2003	2	0	0	0	1	0	0	3
2004	5	0	1	0	0	0	2	8
2005	4	2	0	0	0	0	1	7
2006	4	2	1	0	0	0	1	8
2007	4	1	0	1	0	1	0	7
2008	1	2	0	0	0	0	0	3
2009	1	1	0	0	0	0	1	3
2010	2	0	0	0	0	1	0	3
2011	4	0	0	0	0	0	1	5
2012	2	1	0	0	0	0	0	3
All years	43 (47)	12	2	1	1	2	10	71 (75)

The most common biotype was biotype II: all 43 Hia case isolates belonged to this biotype as well as 2 Hic, and 5 NT-Hi, including the 3 NT isolates typed as ST-23 (also described below). The second most common biotype was biotype I, including 12 Hib, 1 Hie and 2 Hif. There were also 2 biotype III (both NT isolates); 2 biotype IV (1 each of Hid and NT isolate); and 2 biotype V (both NT isolates).

### Population biology of invasive *H. influenzae*

**Fig. 1:**
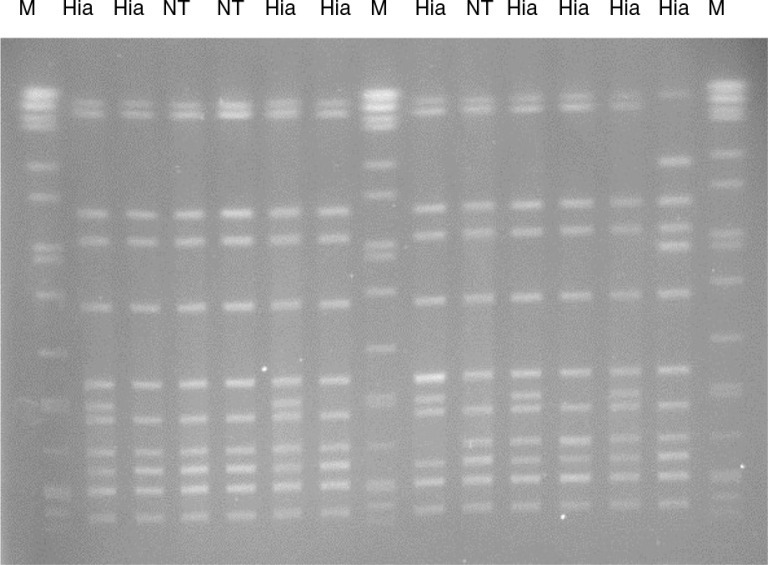
Pulsed-field gel electrophoresis of *SmaI* restricted *Haemophilus influenzae* DNA. M, *Salmonella* Braenderup DNA as size marker; Hia, *Haemophilus influenzae* serotype a; NT, non-typeable *Haemophilus influenzae*.

The encapsulated invasive *H. influenzae* strains appeared to be homogeneous according to their serotypes, with each serotype forming a unique clonal group with little or no sharing of their MLST housekeeping enzyme gene alleles between the different serotypes (Table II). In contrast, 7 of the 10 NT-Hi appeared to be heterogeneous or diverse in their genetic background based on their MLST gene alleles (Table II). The remaining 3 NT-Hi isolates were typed as ST-23, identical to the ST found in Hia. PFGE patterns of these 3 isolates were identical or highly similar to the ST-23 Hia isolates (Fig. 1).

**Table II:** Clonal analysis by multi-locus sequence typing (MLST) of 75 invasive *Haemophilus influenzae* isolated in Nunavut, Canada, 2000–2012

		MLST allelic profile							
			
Serotype	ST	*adk*	*atpG*	*frdB*	*fucK*	*mdh*	*pgi*	*recA*	No. of isolates
a	23	13	16	5	2	3	11	7	47
b	95	31	14	4	5	4	7	9	9
b	635	31	14	4	5	**172**	7	8	2
b	44	**10**	14	4	**3**	4	**3**	8	1
c	9	7	11	6	8	6	16	9	2
d	1288	5	15	7	1	7	5	11	1
e	18	18	6	3	7	10	28	12	1
f	124	22	19	11	11	22	19	15	2
NT	*23*	*13*	*16*	*5*	*2*	*3*	*11*	*7*	*3*^a^
NT	98	14	7	13	15	17	13	1	1
NT	146	11	8	47	7	17	57	41	1
NT	161	41	8	29	9	68	57	8	1
NT	176	5	33	7	15	47	58	29	1
NT	280	3	9	8	2	86	8	4	1
NT	717	5	1	7	32	26	41	29	1
NT	1289	14	51	16	48	49	2	31	1

ST, sequence type.

a Complete loss of capsule synthesis genes. Numbers highlighted in bold represent allelic differences between strains within each serotype.

All the Hia strains examined from Nuvanut did not contain any partial deletion in their capsule synthesis operon involving their IS*1016* elements and *bexA* genes.

### Antibiotic susceptibility of invasive *H. influenzae*

The only antibiotic resistance detected was to ampicillin and it was due to production of β-lactamases. This was found in 8 Hib and 1 NT-Hi.

## Discussion

This study examined the characteristics of invasive *H. influenzae* strains recovered in the post-Hib conjugate vaccine era in a northern territory in Canada. The findings provided a good basis to examine the changes in invasive *H. influenzae* disease since the introduction of the Hib conjugate vaccine. Regardless of including or excluding cases without isolates for the present analysis, Hia appeared to be the predominant serotype found among invasive disease cases in Nunavut (60.6–62.5%, with and without cultures for this study), followed by Hib (16.9–14.8%) and NT strains (14.1–12.5%). In contrast to other studies in urban settings (8,21), only 2 cases due to Hif were found.

Molecular characterization of the invasive *H. influenzae* isolates obtained from Nunavut, Canada, confirmed our previous observations that encapsulated *H. influenzae* are clonal and each serotype appears to be made up of only a single clone with related STs, especially if isolates were obtained from a defined geographical region (22,23). On the national or global scale, some serotypes are known to be made up of more than 1 clone. For example, Hib as well as Hia, in each case has been described to be made up of strains of 2 separate clonal divisions, which can be separated by analytical tools such as multi-locus enzyme electrophoresis or multi-locus sequence typing (24,25). Further, like Hib, most Hia invasive isolates belong to clonal division I (26).

All of the Hia case isolates identified in this study over a period of 13 years belonged to a single sequence type, ST-23. Of interest were another 3 case isolates that were also typed as ST-23 but they did not express any capsular antigens and hence were deemed as NT. Further characterization of these 3 isolates confirmed the complete absence of all capsule synthesis genes. Among our collection of NT-Hi (n=939) archived at the National Microbiology Laboratory (Winnipeg, Manitoba), these were the only NT strains typed as ST-23. PFGE of these 3 ST-23 NT isolates with ST-23 Hia isolates showed almost identical DNA fingerprints suggesting their genetic similarity (Fig. 1). Therefore, it is very likely that these were originally Hia isolates that have lost their capsule synthesis genes at some point, most likely after they were isolated from the patients on culture media.

The capsule of Hia is most similar to Hib. In Hib, the capsule is a polysaccharide based on a disaccharide backbone of ribitol linked to ribitol phosphate (27), while in Hia the backbone disaccharide is glucose and ribitol phosphate (28). The increase in Hia cases in Nunavut did not appear to be related to capsule switching of Hib strains to Hia because the STs of the Hia and Hib case isolates were very different (Table II). Also, the ST-23 Hia isolates identified in Nunavut did not appear to have partial deletion of their capsule synthesis genes, which has been described to be typical of invasive Hib strains (29) and in some Hia strains too (30). Previous studies have also suggested that not all Hia strains have this partial deletion of genes in their capsule synthesis operon (10,31).

Besides their capsule chemistry, another noticeable difference between Hia and Hib may be their susceptibility towards antibiotics with Hib more often found to have resistance to antibiotics like ampicillin while such resistance in Hia is relatively rare. In this study, 8 out of 12 (66.7%) Hib case isolates were found to have resistance to ampicillin due to β-lactamase production versus 0 out of 43 (0%) Hia case isolates. One hypothesis may be that unlike Hib which have been in circulation for a long time and have acquired antibiotic resistance genes including those that encode for β-lactamases (32), Hia is a recently introduced pathogen as suggested to be the case in Alaska, USA (31), and therefore, have not acquired antibiotic resistance genes. Indeed, this observation of a difference between Hia and Hib in terms of their susceptibility towards commonly prescribed antibiotics had been confirmed with a larger sample size collected from 3 Canadian provinces over a period of 2 decades (33). If indeed the differential susceptibility of Hia and Hib towards commonly prescribed antibiotics depends on the longevity of the strain circulating in a population, then it is quite possible that as Hia circulates in a population over time, it will pick up antibiotic resistance genes and become resistant to antibiotics. Based on the success of the Hib conjugate vaccine, it is reasonable to assume that a Hia conjugate vaccine would protect against Hia infection and carriage as well as minimize its opportunity to pick up antibiotic resistance genes.

Given the severity of illness due to Hia (34,35), sporadic and recurrent Hia infections as well as potential Hia outbreaks (31,36,37) pose a significant threat. In addition, the lack of a vaccine and the lack of published guidelines for the prophylaxis of contact exposure may represent a gap in our public health system for an emerging pathogen in a vulnerable population. Therefore, further population-based surveillance studies to identify those who may be at risk of this infection may help us to understand the epidemiology of invasive Hia infections as well as to pave the way for potentially developing a Hia monovalent or Hia–Hib bivalent vaccine for the protection of populations at risk of exposure to this pathogen (38,39).
